# Gypenosides regulate farnesoid X receptor-mediated bile acid and lipid metabolism in a mouse model of non-alcoholic steatohepatitis

**DOI:** 10.1186/s12986-020-00454-y

**Published:** 2020-05-01

**Authors:** Hongshan Li, Yingfei Xi, Xin Xin, Huajie Tian, Yiyang Hu

**Affiliations:** 1 Institute of Liver Disease, Shuguang Hospital, Shanghai University of Traditional Chinese Medicine, Shanghai, China; 2 Liver Disease Department, Hwa Mei Hospital, University of Chinese Academy of Sciences, Ningbo, Zhejiang China; 3 Key Laboratory of Diagnosis and Treatment of Digestive System Tumors of Zhejiang Province, Ningbo, 315010 Zhejiang China; 4 Medical School of Ningbo University, Ningbo, Zhejiang China

**Keywords:** Gypenosides, Farnesoid X receptor, Bile acid, Lipid metabolism

## Abstract

**Background:**

Gypenosides (Gyp) are the main ingredient of the Chinese medicine, *Gynostemma pentaphyllum.* They are widely used in Asia as a hepatoprotective agent. Here, we elucidated the mechanism of Gyp in non-alcoholic steatohepatitis (NASH) with a focus on farnesoid X receptor (FXR)-mediated bile acid and lipid metabolic pathways.

**Methods:**

NASH was induced in mice by high-fat diet (HFD) feeding, while mice in the control group were given a normal diet. At the end of week 10, HFD-fed mice were randomly divided into HFD, HFD plus Gyp, and HFD plus obeticholic acid (OCA, FXR agonist) groups and were given the corresponding treatments for 4 weeks. Next, we analyzed the histopathological changes as well as the liver triglyceride (TG) level and serum alanine aminotransferase (ALT), aspartate aminotransferase (AST), fasting blood glucose (FBG), fasting insulin (FINS), TG, total cholesterol (TC), and low-density lipoprotein cholesterol (LDL-C) levels as well as the bile acid profile. We carried out RT-PCR and western blotting to detect HFD-induced alterations in gene/protein expression related to bile acid and lipid metabolism.

**Results:**

The HFD group had histopathological signs of hepatic steatosis and vacuolar degeneration. The liver TG and serum ALT, AST, FBG, FINS, TC, and LDL-C levels as well as the total bile acid level were significantly higher in the HFD group than in the control group (*P* < 0.01). In addition, we observed significant changes in the expression of proteins involved in bile acid or lipid metabolism (*P* < 0.05). Upon treatment with Gyp or OCA, signs of hepatic steatosis and alterations in different biochemical parameters were significantly improved (*P* < 0.05). Further, HFD-induced alterations in the expression genes involved in bile acid and lipid metabolism, such as CYP7A1, BSEP, SREBP1, and FASN, were significantly alleviated.

**Conclusions:**

Gyp can improve liver lipid and bile acid metabolism in a mouse model of NASH, and these effects may be related to activation of the FXR signaling pathway.

## Background

Non-alcoholic fatty liver disease (NAFLD) is a complication of obesity and metabolic syndromes [1]. The clinical presentation of NAFLD ranges from simple steatosis and non-alcoholic fatty liver to hepatic fibrosis [2]. Hepatic fibrosis and non-alcoholic steatohepatitis (NASH) are among the main reasons for the development of liver cirrhosis and hepatocellular carcinoma [3, 4].

Along with increased obesity rates, the global prevalence of NAFLD has reached up to 24%, creating a huge economic burden on healthcare systems worldwide [5]. Therefore, implementing strategies that lead to the prevention and treatment of NASH and NAFLD will have a significant socioeconomical impact. To date, dietary adjustments and lifestyle changes are the standard treatment strategies for NASH, while efficient therapeutic agents are not yet available in clinical practice [6].

*Gynostemma pentaphyllum* (Thunb.) Makino, is a perennial climbing plant of the genus Cucurbitaceae [7]. In China and other Asian countries, *Gynostemma pentaphyllum* has been widely used for its beneficial pharmacological effects, such as regulating blood lipid and sugar levels as well as anti-inflammatory, hepato-protective, anti-tumor, and immunomodulatory activities [8–11]. The pharmacological impact of *Gynostemma pentaphyllum* is attributed to the main ingredient, gypenosides (Gyp) [12, 13]. We previously demonstrated that Gyp can be used to treat NASH via the regulation of lipid metabolism [14]. However, their therapeutic impact and mechanism of action require further validation.

Farnesoid X receptor (FXR), a nuclear receptor superfamily member, is important for bile acid and glycolipid metabolism. Kim et al. previously suggested that FXR is a potential target for NAFLD treatment [15]. Moreover, Neuschwander-Tetri et al. demonstrated that a FXR agonist, obeticholic acid (OCA), can significantly improve the pathological outcomes of NASH and thus can be used as a potential treatment [16]. Mounting evidence confirmed that FXR agonists can improve insulin resistance and regulate the glycolipid metabolism [17, 18]. Interestingly, we previously showed that Gyp have beneficial effects in NASH via the improvement of lipid metabolism [14]. Therefore, we used a mouse model of high-fat diet (HFD)-induced NASH to gain mechanistic insights into the impact of Gyp in NASH. Further, we aimed to explore possible crosstalk between Gyp and the FXR-mediated lipid and bile acid metabolic pathways.

## Methods

### Laboratory animals and experimental design

A total of 32 male C57BL/6 specific-pathogen free (SPF) mice, weighing 16–20 g, were purchased from the Nanjing Biomedical Research Institute, Nanjing University (license number: SCXK (Su) 2015–0001). For an acclimation period of 1 week, mice were housed in the animal center of Ningbo University under SPF conditions with food and water available ad libitum. Following the acclimation period, mice were randomly assigned to a control group (*n* = 8 mice) and an experimental group (*n* = 24 mice). Next, mice in the experimental group were fed with a HFD (Research Diets, D12492i, 60% Kcal fat, energy density: **5.21** kcal/g, New Brunswick, NJ, USA) for 14 consecutive weeks, while mice in the control group were given a normal control diet (Research Diets, D12450B, 10% Kcal fat, energy density: **3.82** kcal/g, New Brunswick, NJ, USA) for 14 consecutive weeks [19]. The ingredients of the normal control diet and HFD are presented in Table 1. At the end of week 10, mice in the experimental group were randomly divided into a HFD group, HFD plus Gyp group, and HFD plus OCA group (*n* = 8/group). In the HFD plus Gyp group, mice were given a daily dose of 100 mg/kg Gyp (Shanghai Ronghe Pharmaceutical Technology Development Co., Ltd., China; batch number: 171019), for 4 consecutive weeks (from week 11 to week 14) as described previously [20]. The fingerprint spectrum of Gyp was analyzed by ultra high-performance liquid chromatography (Thermo Scientific, USA) and quadrupole/electrostatic field orbit trap high-resolution mass spectrometry. Sample preparation and analysis were performed as described previously [21].

**Table 1:** Ingredients of the normal and high-fat diets

Ingredient	Normal control diet (g/kg)	High-fat diet (g/kg)
Casein, Lactic, 30 Mesh	200	200
Cystine, L	3	3
Lodex 10	35	125
Sucrose, Fine Granulated	354	72.8
Starch, Corn	314	–
Solka Floc, FCC200	50	50
Lard	20	245
Soybean Oil, USP	25	25
S10026B	50	50
Choline Bitartrate	2	2
V1001C	1	1
Dye, Blue FD&C #1, Alum. Lake 35–42%	0.05	0.05

Notes: The energy densities of the normal control diet and high-fat diet were 3.82 kcal/g and 5.21 kcal/g, respectively

In the HFD plus OCA group, mice were given 10 mg/kg/day OCA (BioVision, USA; batch number: 3J28B18980) via oral gavage for 4 consecutive weeks as described previously [22]. Mice in the HFD and control groups received equal volumes of drinking water via oral gavage. The experimental protocol was revised and approved by the Experimental Animal Ethics Committee of Ningbo University. All procedures performed in studies involving animals were in accordance with the ethical standards of the institution or practice at which the studies were conducted.

### Specimen collection

At week 14, mice were fasted for 12 h and anesthetized with an intraperitoneal injection of 3% sodium pentobarbital (3 mL/kg), and different specimens were collected as described previously [14]. Briefly, blood was collected from the inferior vena cava and centrifuged, and serum samples were collected and stored at − 70 °C until the detection of biochemical biomarkers. Two liver tissue specimens were extracted from the same liver lobe and placed in either 10% neutral formalin buffer and paraffin embedded for hematoxylin and eosin (HE) staining or frozen in optimal cutting temperature (OCT) compound for oil red O staining. Another liver tissue specimen was collected from each mouse and stored at − 70 °C for subsequent RT-PCR and western blot analyses. The total mouse body weight and liver wet weight were recorded, and the liver index was calculated according to the following formula: liver index = liver wet weight/body weight * 100%.

### Histopathological examination

HE staining was performed on 4-μm-thick sections according to the standard published protocols [23]. Sections were examined by experienced liver pathologists. The NAFLD activity score (NAS) was determined as described previously according to the following score [24]: steatosis (0 = < 5%; 1 = 5–33%; 2 = 33–66%; 3 = > 66%); intralobular inflammation (0 = no lesions; 1 = < 2 lesions/field of view; 2 = 2–4 lesions/field of view; 3 = > 4 lesions/field of view; and ballooning degeneration (0 = none; 1 = rare new balloon cells; 2 = common new balloon cells).

Frozen liver tissues were sectioned at 10-μm thickness on a cryostat (Leica CM1850, Germany), and fat cells were detected by an oil red O staining kit (Nanjing Jiancheng Bioengineering Institute, Jiangsu, China; batch number: 20180704) following the manufacturer’s instructions. Next, sections were analyzed under an inverted fluorescence microscope (Leica 37XB, Germany) and photographed.

### Biochemical parameters

The liver triglyceride (TG) content was analyzed with a TG assay kit (Zhejiang Dongou Diagnostics Co. Ltd. Zhejiang, China, batch number: 201804007) according to the manufacturer’s protocols. Specimen preparation and homogenization were carried out as previously detailed [14].

Serum alanine aminotransferase (ALT), aspartate aminotransferase (AST), and fasting blood glucose (FBG) levels were detected using standard kits according to the manufacturer’s protocols (Nanjing Jiancheng Bioengineering Institute, Jiangsu, China; batch numbers: 20180629, 20180629 and 20180402137, respectively).

A serum TG kit was obtained from Zhejiang Dongou Diagnostics Co. Ltd. (batch number: 201004007; Zhejiang, China). Kits for the detection of total cholesterol (TC; batch number: 20180526), low-density lipoprotein cholesterol (LDL-C; batch number: 20180705), and high-density lipoprotein cholesterol (HDL-C; batch number: 20180706) were purchased from Nanjing Jiancheng Bioengineering Institute (Jiangsu, China). All parameters were detected following the manufacturers’ protocols on a microplate reader (BioTek, MQX200R).

The fasting insulin (FINS) levels were detected in mice sera samples using a mouse insulin enzyme-linked immunosorbent assay (ELISA) kit (Crystal Chem, Inc., IL, USA; batch number: 18 MAUMI477A and cat. no. 90080) following the manufacturer’s instructions. The absorbance was measured within 30 min, and the insulin concentration in each sample was calculated using a standard curve. For each mouse, the homeostasis model assessment-insulin resistance (HOMA-IR) index was estimated using: HOMA-IR = FBG × FINS/22.5 [25].

### Quantitative bile acid profiling

A total of 10 mg liver tissue was cut and transferred to a microcentrifuge tube. Also, 25 mg beads and 20 μl ultrapure water were mixed using a homogenizer (BB24, Next Advance, Inc., Averill Park, NY, USA). Samples were mixed with 180 μl acetonitrile/methanol (8:2) containing 10 internal standards and centrifuged at 13000 rpm for 20 min and 4 °C (Microfuge 20R, Beckman Coulter, Inc., Indianapolis, IN, USA). Following centrifugation, the supernatant was transferred to a 96-well plate and freeze-dried with FreeZone freeze dryer (Labconco, Kansas City, MO, USA). The dried sample powder and freeze-dried standard were remixed with 1:1 (v/v) acetonitrile/methanol (80/20, v/v) and ultrapure water, and then centrifuged at 13000 rpm for 20 min and 4 °C (Microfuge 20R). A total of 5 μl supernatant was transferred to a 96-well plate and then subjected to LC-MS analysis.

Bile acids were analyzed using an ultra-performance liquid chromatography tandem mass spectrometry (UPLC-MS/MS) system (ACQUITY UPLC-Xevo TQ-S, Waters Corp., Milford, MA, USA), in accordance with previous reports [26, 27].

### Real-time quantitative polymerase chain reaction

Total RNA was extracted from the frozen liver tissues using an RNA extraction kit (Sangon Biotech Co., Ltd., China; batch number: E928KA9723) following the manufacturer’s instructions. The RNA concentration was estimated at 260 nm on an Infinite 200 PRO microplate reader (Tecan Group Ltd., Switzerland). Different PCR primers were designed by BioTNT (Shanghai, China) (Table 2). Reverse transcription was achieved using an iScript™ cDNA Synthesis Kit (Bio-Rad, Hercules, CA, USA; cat. no. 170–8891). Real-time PCR was performed using a TB Green™ Premix Ex Taq™ (TaKaRa, Janpan, cat. no. RR420A) and a QuantStudio™ real-time PCR system (Applied Biosystem, Foster City, CA, USA). β-Actin served as the internal control.

**Table 2:** Primer sequences used for real-time polymerase chain reaction

Gene	Gene ID	Forward primer (5′ to 3′)	Reverse primer (5′ to 3′)
FXR	20186	CAGATTTCCTCCTCGTCTTAC	CCTGAGTTCATAGATGCCA
SHP	23957	TTCCTTGCTTTGGATACAGTG	GAGGTTTGGGGAGTCATCA
SREBP1	20787	GGCTTGGTGATGCTATGTTGA	GGCAAAGGAACAACTGAGACC
FASN	14104	GTGTGGTGGGTT TGG TGA AT	AGATGTGTTGCT GAG GTT GGA
SCD1	20249	TGAGGG GCA GTG TCT GTA A	GGAAACTAAAGCAATGGA TGG
PPARα	19013	GAGGATGGGGACTTTTGTTCT	GGC TTT TTG GCT GTA GGA GG
CPT1	12894	ACC CAG TCA GAT TCC AAC C	ACAAAGCACCCA TTA CTT GAG
MTTP	17777	CCACCAGAATCGTAAGGTTCA	GGACAGCAGGAT GTT CTT CAC
CYP7A1	13122	GTACCTTGATGAAAGTGGGAA	ATTGCT TGA TTT CTT GGA CAG
FGFR4	14186	CCT TCT GTT CCA GCC TTA TG	TTC TCT GAG GAT GAG TCC AAG
BSEP	27413	AAT GTT CAG TTC CTC CGT TC	GTC CCC ATA CTT GAT GTT GTC
NTCP	20493	GTCATCAATGTGGGCAACAGC A	TGAAAGGCATCAGGGAGGAGG TA
KLB	83379	CCT TCT GTT CCA GCC TTA TG	TTC TCT GAG GAT GAG TCC AAG
FGF15	14170	GAAGCCAGA AGG TAT GAA GTC	CCA AGT TTG TAA CCC CAG T
β-Actin	81822	CCT CTA TGC CAA CAC AGT	AGC CAC CAA TCC ACA CAG
LPL	16956	CGA GAGCGAGAA CAT TCC CT	TGT CCA CCT CCG TGT AAA TCA A

*FXR* farnesoid X receptor, *SHP* small heterodimer partner, *SREBP1* sterol-regulatory element-binding protein 1, *SCD1* stearyl coenzyme A desaturation enzyme 1, *FASN* fatty acid synthetase, *PPARα* peroxisome proliferator-activated receptor alpha, *CPT1* carnitine palmitoyl transferase 1, *MTTP* microsomal triglyceride transfer protein, *CYP7A1* cholesterol 7-alpha hydroxy-lase, *FGFR4* fibroblast growth factor receptor 4, *BSEP* bile salt export protein, *NTCP*, Na+-taurocholate co-transporting polypeptide, *KLB* Klotho beta, *FGF15* fibroblast growth factor 15, *LPL* lipoprotein lipase

### Western blotting

Total proteins were extracted from 50 mg of liver tissue with 600 μl radioimmunoprecipitation assay (RIPA) lysis buffer (Beyotime,China; cat. no. P0013B) supplemented with protease and phosphatase inhibitors (Beyotime; cat. no. P1045–1 and P1045–2, respectively). The mixture was homogenized twice at 65 Hz for 1 min on an automatic sample rapid grinder (Shanghai Jingxin Industrial Development Co., Ltd. Company, model: JXFSTPR-24). Next, the mixture was centrifuged at 12000 rpm for 15 min at 4 °C, and the middle layer was extracted with a 1-ml syringe. The upper fat layer and the pellet were discarded. The protein concentration was estimated using a Pierce™ BCA Protein Assay Kit (Thermo Fisher, cat. no. TI269557).

Proteins were separated using 10% or 12% sodium dodecyl sulfate polyacrylamide gel electrophoresis (SDS-PAGE) gels and sequentially transferred onto Immobilon-FL Transfer membranes. Following blocking with blocking buffer (Odyssey, cat no. 927–40,000), membranes were incubated with the primary antibodies at 4 °C overnight. The following primary antibodies were used in this study: anti-FXR monoclonal antibody (Thermo Fisher, A9033A; 1:1000); anti-NROB2 antibody (Abcam, Cambridge, MA, USA; ab186874;1:500); anti-CYP7A1 antibody (Abcam, ab65596, 1:1000); anti-bile salt export pump (BSEP) polyclonal antibody (Thermo Fisher, PA5–78690, 1:2000); anti-Na^+^-taurocholate cotransporting polypeptide (NTCP) polyclonal antibody (Thermo Fisher, PA5–80001, 1:2000); anti-fibroblast growth factor 15 (FGF15) antibody (Abcam; ab229630, 1:1000); anti-FGF receptor 4 (FGFR4) antibody (Abcam, ab119378,1:500); anti-Klotho beta (KLB) antibody (Abcam, ab233416, 1:1000); anti-sterol regulatory element-binding protein 1 (SREBP1) antibody (Abcam, ab28481, 1:1000); anti-SCD1 antibody (Abcam,ab19862, 1:1000); anti-fatty acid synthetase antibody (Abcam, ab22759, 1:1000); anti-peroxisome proliferator-activated receptor alpha (PPARα) antibody (Abcam, ab61182, 1:500); anti-carnitine palmitoyl transferase 1A (CPT1A) antibody (Abcam, ab128568, 1:1000); anti-lipoprotein lipase (LPL) antibody (Abcam, ab21356, 1:1000); anti-microsomal triglyceride transfer protein (MTTP) antibody (Abcam, ab75136, 1:500), and anti-glyceraldehyde-3-phosphate dehydrogenase (GAPDH) rabbit polyclonal antibody (Proteintech Group, Inc., 10,494–1-AP, 1:1000). The next day, membranes incubated with horseradish peroxidase (HRP)-conjugated goat anti-mouse IgG (Beijing Dingguo Changsheng Biotechnology Co., IH-0031) or HRP-conjugated goat anti-rabbit IgG (Beijing Dingguo Changsheng Biotechnology Co., IH-0011) for 1 h at room temperature. BeyoECL Plus A (Beyotime, P0018 M-1) and BeyoECL Plus B (Beyotime, P0018 M-2) were used for development, and chemiluminescence was used for visualization. Chemographic analysis software (ChemiScope 3000 mini, Shanghai Qinxiang Scientific Instrument Co., Ltd) was used to analyze the grey values of each band.

### Statistical analysis

The data are presented as the mean ± standard deviation. One-way analysis of variance (least significance difference [*LSD*] test) was used to compare the data for different groups. All statistical analyses were performed using SPSS software version 16.0 (SPSS, Inc., Chicago, IL USA). A *P* value < 0.05 was considered to be statistically significant.

## Results

### Dietary intake and activity level of mice

Our results demonstrated that the dietary intake of mice in the HFD group was higher than that of mice in control group (*P* < 0.01). There were no significant differences in dietary intake among the HFD group, Gyp-treated group and OCA-treated group (*P* > 0.05; Table 3).

**Table 3:** Dietary intake of mice among the different experimental groups (kcal (each mouse^− 1^/day))

Group	***n***	11 week	12 week	13 week	14 week
C	8	8.94 ± 0.16	8.85 ± 0.21	9.21 ± 0.25	8.95 ± 0.13
HFD	8	12.20 ± 0.22**	12.10 ± 0.39**	12.41 ± 0.25**	12.24 ± 0.28**
HFD + Gyp	8	12.10 ± 0.34	12.00 ± 0.38	12.32 ± 0.13	12.25 ± 0.20
HFD + OCA	8	12.02 ± 0.23	12.09 ± 0.38	12.42 ± 0.36	12.18 ± 0.21

**P < 0.01 vs C group. *C* control, *HFD* high-fat diet, *Gyp* gypenosides, *OCA* obeticholic acid

Further, during the experimental phase, we observed a phenomenon that mice in the HFD group were relatively lazy and showed reduced activity compared to mice in the control group. On the other hand, the mice in the Gyp-treated group and the OCA-treated group were relatively active compared to mice in the HFD group.

### Analysis of gyp

**Fig. 1:**
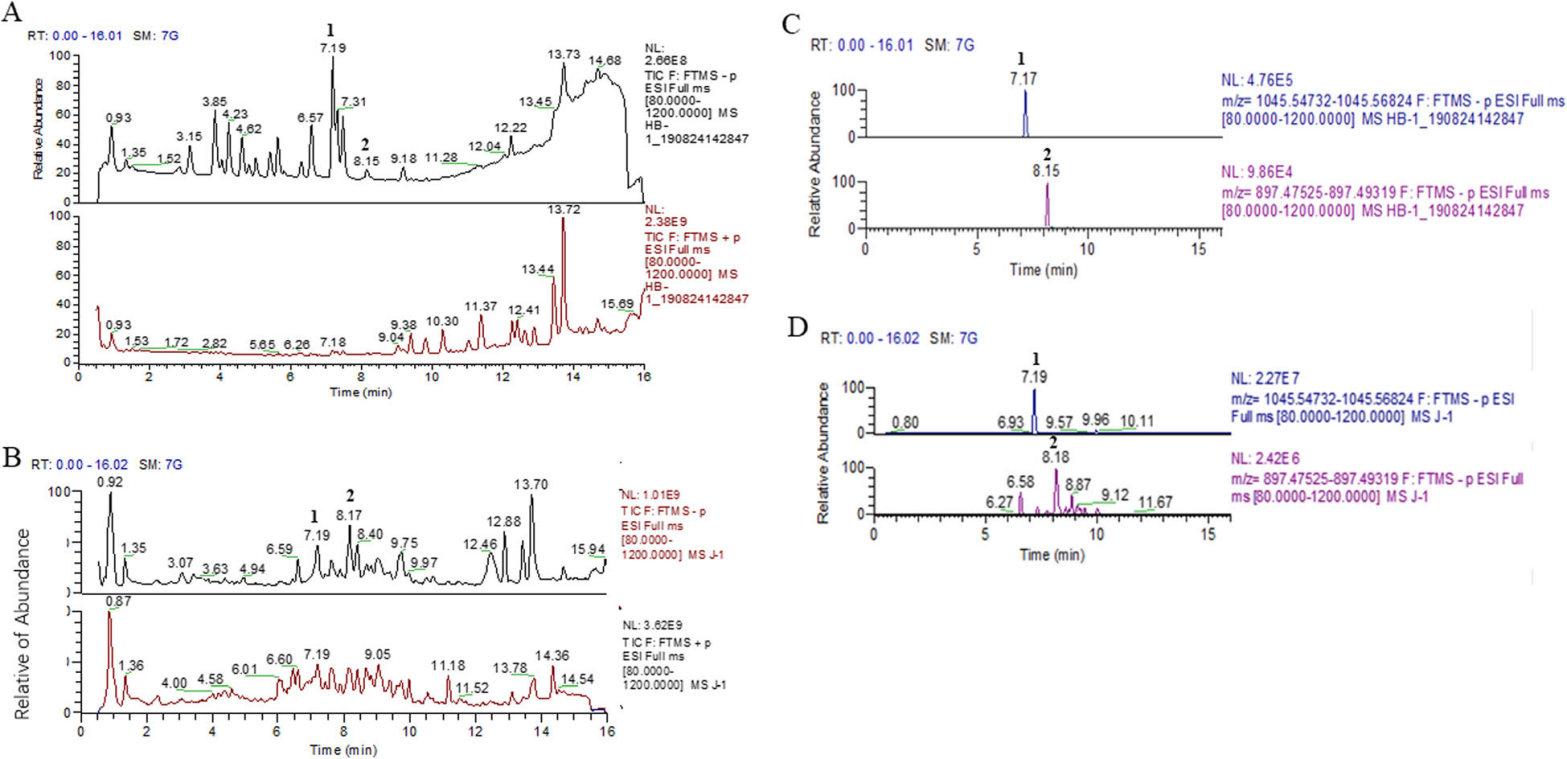
Total-ion chromatograms and select-ion chromatograms of reference standard and gypenosides. **a** Total-ion chromatograms of reference standard; **b** Total-ion chromatograms of gypenosides sample; **c** Select-ion chromatograms of reference standard; **d** Select-ion chromatograms of gypenosides sample; 1, gypenosides XLIX; 2, Gypenosides A

High-performance liquid chromatography and mass spectrometric analysis of the Gyp fingerprint spectrum revealed that the contents of gypenoside XLIX (molecular formula: C52H86O21, molecular weight: 1047.23) and gypenoside A (molecular formula: C46H74O17, molecular weight: 898.49) were 574.91 mg/g and 342.66 mg/g, respectively (Fig. 1).

### Impact of gyp treatment on the liver index and mice weight

**Fig. 2:**
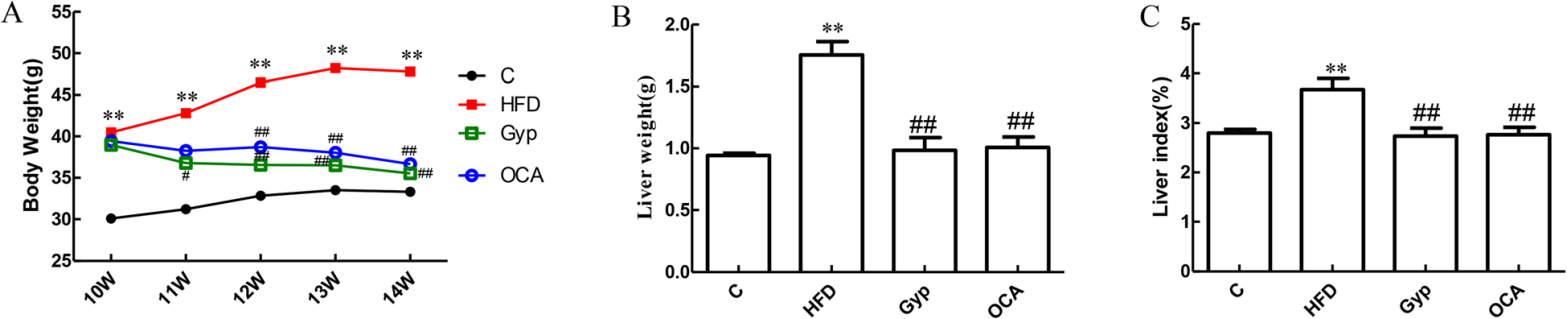
Alterations in body weight, liver wet weight, and liver index in the different experimental groups. **a** Body weight, **b** liver wet weight, and **c** liver index. ** *P* < 0.01, vs control group; ^##^*P* < 0.01, ^#^*P* < 0.05, vs HFD group. C, control; HFD, high-fat diet; Gyp, gypenosides; OCA, obeticholic acid

Compared to the control group, mice in the HFD group had a significantly higher body weight starting from weeks 10–14 (Fig. 2a, *P* < 0.01). In the Gyp- and OCA-treated groups, the mice weight was significantly lower than that in the HFD group at week 12 (Fig. 2a, *P* < 0.01). Interestingly, there was no significant difference in body weight between the Gyp group and the OCA-treated group (Fig. 2a, *P* > 0.05).

The liver wet weight and liver index in the HFD group were significantly higher than those in the control group (*P* < 0.01). In the Gyp-treated and the OCA-treated groups, the wet weight of livers as well as the liver indices were significantly lower than those of the HFD group (Fig. 2b and c, *P* < 0.01). There were no significant differences in the liver wet weight and liver index between the Gyp-treated group and the OCA-treated group (Fig. 2b and c, *P* > 0.05).

### Gyp treatment improved hepatic steatosis

**Fig. 3:**
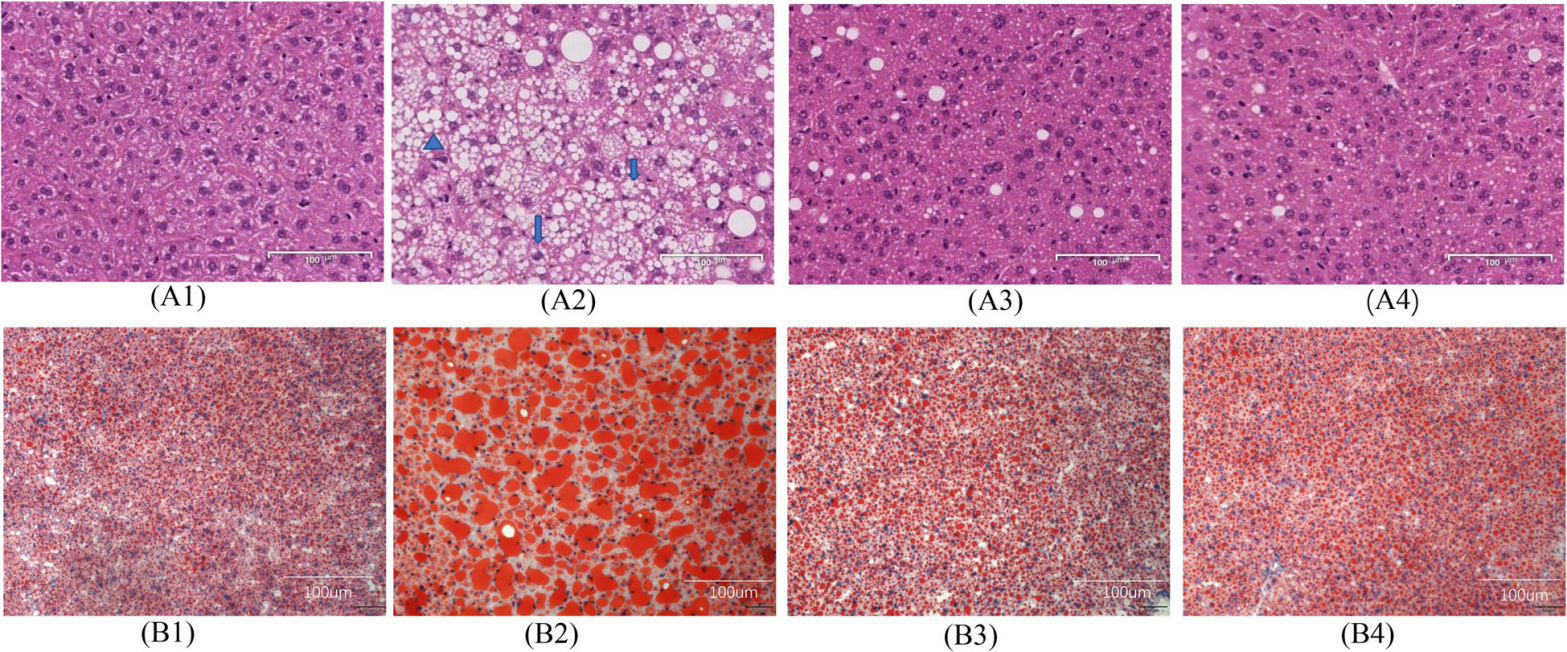
Representative images of pathological changes in liver tissues among the different experimental groups. A: Hematoxylin and eosin staining, B: Oil red O staining. 1: Control group, 2: high-fat diet group, 3: gypenosides group, 4: obeticholic acid group, scale bar =100 μm, *n* = 8. The triangular shape indicates steatosis, and an arrow indicates a balloon-like change

In control mice, HE staining revealed typical liver histology without signs of inflammation or accumulation of lipid droplets in the hepatocytes (Fig. 3A1). Following HFD feeding, mice in the HFD group demonstrated signs of obvious hepatic steatosis accompanied by scattered inflammation and ballooning degeneration (Fig. 3A2). Particularly, hepatocytes appeared swollen with a large number of lipid droplets accumulated in the cytoplasm. Similarly, oil red O staining verified the accumulation of large lipid droplets in the hepatocytes with dark red intracellular staining in the HFD group mice (Fig. 3B2). These results confirmed that HFD feeding for 14 weeks induced NASH. These pathological changes were significantly alleviated after treatment with Gyp or OCA (Fig. 3A3 and A4, respectively). Further, oil red O staining confirmed the alleviation of hepatic steatosis signs in the Gyp-treated group and the OCA-treated group (Fig. 3B3 and B4, respectively).

**Fig. 4:**
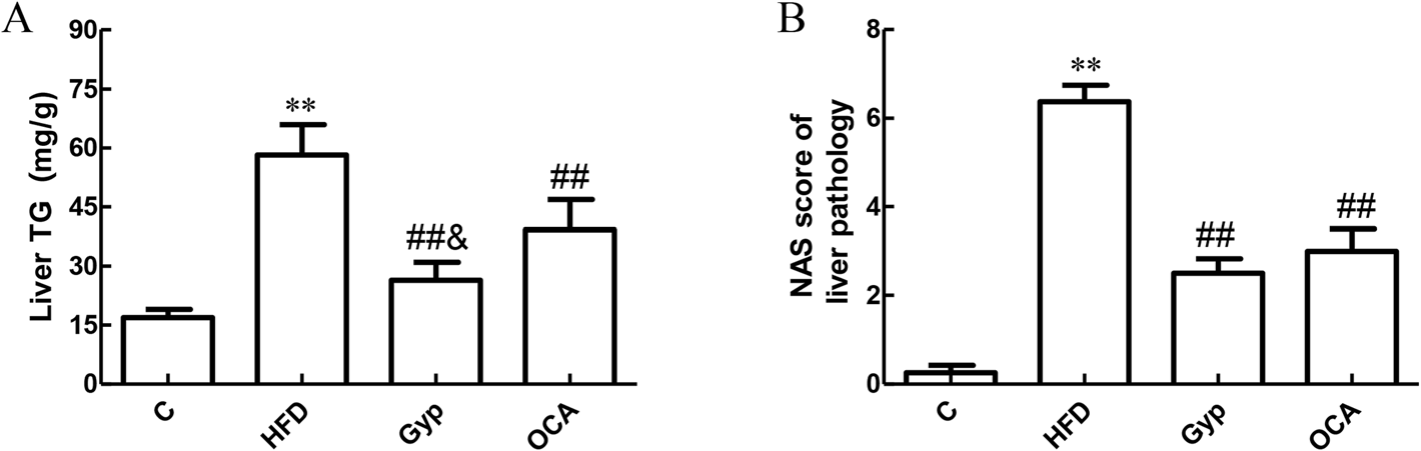
Changes in liver TG and the NAS among the different experimental groups: **a** Liver TG, **b** NAS score. **P < 0.01, vs control group; ^##^*P* < 0.01, ^#^*P* < 0.05, vs HFD group; ^&^*P* < 0.05, vs the OCA group, *n* = 8. TG, triglycerides; NAS, NAFLD activity score; C, control; HFD, high fat diet; Gyp, gypenosides; OCA, obeticholic acid

Next, we examined the liver TG levels and NAS scores among mice from the different experimental groups. Compared to the control group, mice in the HFD group had significantly higher liver TG levels and NAS scores (Fig. 4a and b, *P* < 0.01). In the Gyp-treated and OCA-treated groups, the liver TG and NAS scores were significantly lower than those in the HFD group (Fig. 4a and b, *P* < 0.01). Interestingly, the liver TG levels were significantly lower in the Gyp-treated group than in the OCA-treated group (Fig. 4a, *P* < 0.05), while the difference in NAS scores was not significant (Fig. 4b, *P* > 0.05).

### Gyp treatment improved serum biochemical parameters

**Fig. 5:**
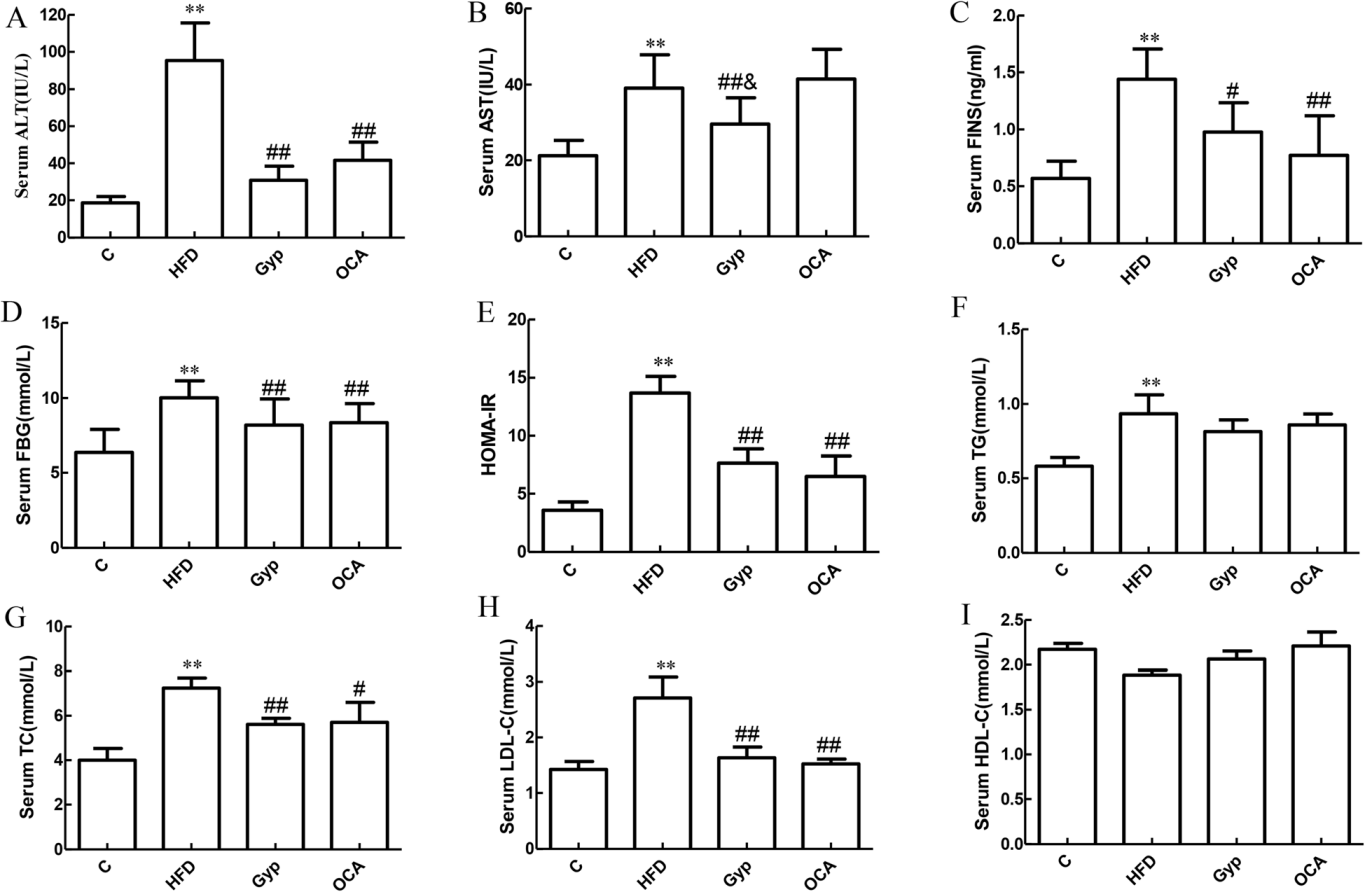
Bar charts showing the changes in the different biochemical assays among the experimental groups. **a** ALT activity, **b** AST activity, **c** FINS level, **d** FBG level; **e** HOMA-IR, **f** TG level, **g** TC level, **h** LDL-C level, and **i** HDL-C level. **P < 0.01, vs control group; ^##^*P* < 0.01, ^#^*P* < 0.05, vs HFD group; ^&^*P* < 0.05, vs OCA group. Data are presented as mean ± standard deviation. *n* = 8, ALT, alanine aminotransferase; AST, aspartate aminotransferase; FINS, fasting insulin; FBG, fasting blood glucose; HOMA-IR, Homeostatic Model Assessment for Insulin Resistance; TG, triglycerides; TC, total cholesterol; LDL-C, low-density lipoprotein cholesterol; HDL-C, high-density lipoprotein cholesterol; C, control; HFD, high-fat diet; Gyp, gypenosides; OCA, obeticholic acid

We examined the impact of HFD feeding and Gyp treatment on different biochemical parameters in serum samples. In the HFD group, serum ALT and AST levels were significantly higher than those of the control group (Fig. 5a and b, *P* < 0.01). Treatment with Gyp significantly decreased the serum ALT and AST activities, while treatment with OCA significantly decreased the serum ALT activities (Fig. 5a and b, *P* < 0.05). Moreover, compared with the control diet, HFD feeding for 14 weeks caused a significant increase in the serum FINS and FBG levels as well as the HOMA-IR index (Fig. 5c-e, *P* < 0.01). In the treated groups, the serum FINS and FBG levels as well as the HOMA-IR were significantly lower in the Gyp-treated and OCA-treated groups compared with the HFD group (Fig. 5c-e, *P* < 0.01). Notably, no significant differences were observed in the serum FINS and FBG levels or the HOMA-IR between the Gyp-treated and OCA-treated groups (Fig. 5c-e, *P >* 0.05).

In the HFD group, serum TG, TC, and LDL-C levels were significantly higher than those of the control group (Fig. 5f-h, *P* < 0.01). Additionally, the serum TC and LDL-C levels were significantly lower in the Gyp- and OCA-treated groups compared to the HFD group (Fig. 5g and h, *P* < 0.05). There were no significant differences in the TG levels between the Gyp and OCA groups (Fig. 5f) or in the HDL-C levels among the examined groups (Fig. 5i).

### Gyp treatment influenced the bile acid profile

**Fig. 6:**
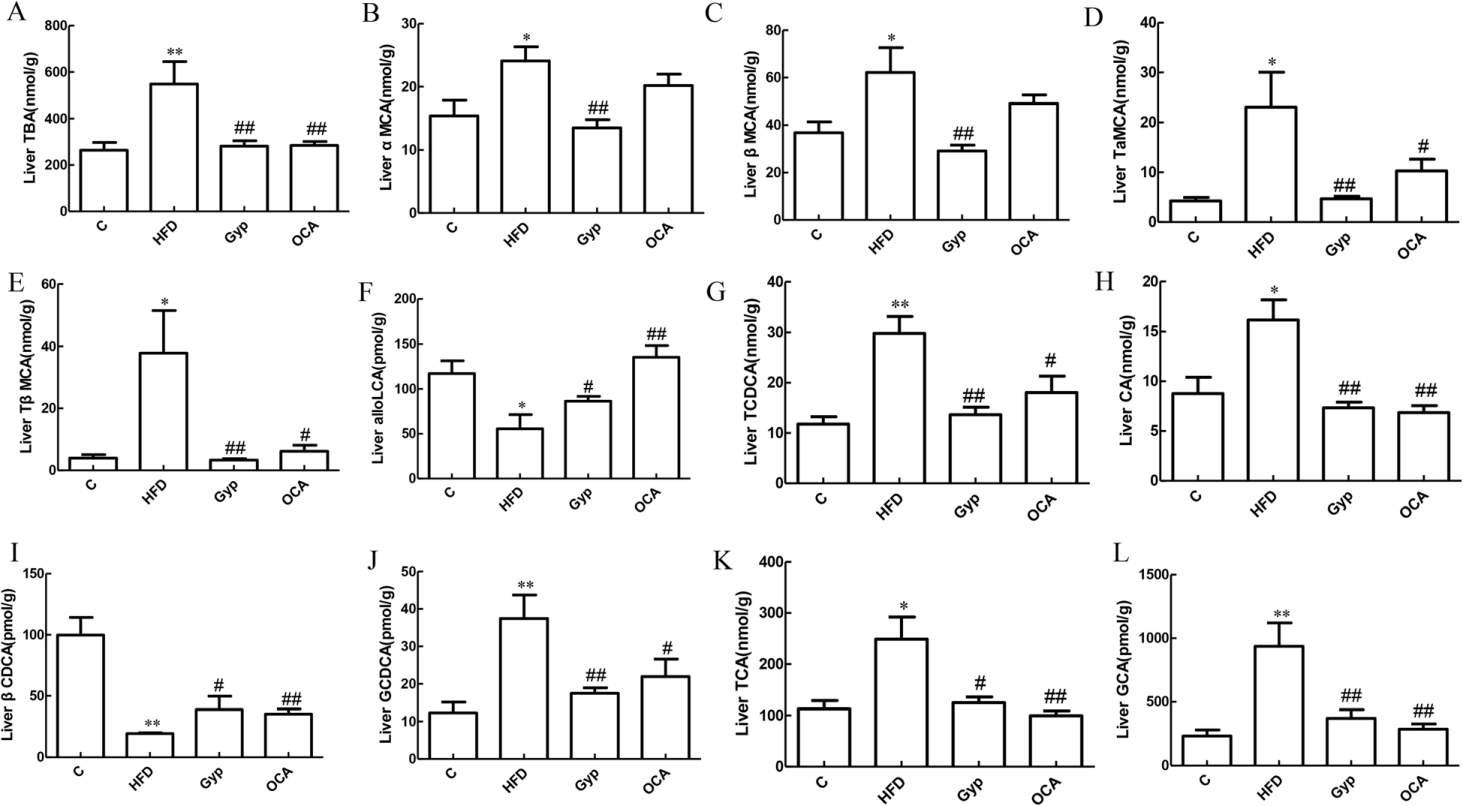
Evaluation of liver bile acid content among the different experimental groups. **a** TBA, **b** αMCA, **c** βMCA, **d** TαMCA, **e** TβMCA, **f** alloLCA, **g** TCDCA, **h** CA, **i** βCDCA, **j** GCDCA, **k** TCA, and **l** GCA. **P < 0.01, *P < 0.05, vs control group; ^##^*P* < 0.01, ^#^*P* < 0.05, vs HFD group; ^&^*P* < 0.05, vs OCA group. Data are expressed as mean ± standard deviation, *n* = 4–7. TBA, total bile acid; αMCA, α-muricholic acid; βMCA, β-muricholic acid; TαMCA, tauro α-muricholic acid; TβMCA, tauro β-muricholic acid; alloLCA, allolithocholic acid; TCDCA, taurochenodeoxycholic acid; CA, cholic acid; βCDCA, 3β-chenodeoxycholic acid; GCDCA, glycochenodeoxycholic acid; TCA, taurocholic acid; GCA, glycocholic acid; C, control; HFD, high-fat diet; Gyp, gypenosides; OCA, obeticholic acid

Next, we analyzed the differences in bile acid content among mice in the different experimental groups. Compared with those in the control group, the levels of the total bile acid (TBA), α-muricholic acid (αMCA), tauro α-muricholic acid (TαMCA), β-muricholic acid (βMCA), tauro β-muricholic acid (TβMCA), taurochenodeoxycholic acid (TCDCA), cholic acid (CA), glycochenodeoxycholic acid (GCDCA), glycocholic acid (GCA), and taurocholic acid (TCA) were significantly increased in liver tissues from the HFD group (Fig. 6a-e, g, h and j-l, respectively; *P* < 0.05), while alloLCA and βCDCA levels were significantly decreased (Fig. 6f and i, respectively; *P* < 0.05). Compared with those in the HFD group, the TBA, TaMCA, TβMCA, TCDCA, CA, GCDCA, GCA, and TCA levels were significantly lower in the Gyp-treated and OCA-treated groups (Fig. 6a, d, e, g, h and j-l, *P* < 0.05). In addition, the liver contents of aMCA and βMCA were significantly lower in the Gyp-treated group than in the HFD group (Fig. 6b and c, *P* < 0.05). On the other hand, the alloLCA and βCDCA levels in the Gyp-treated and OCA-treated groups were significantly higher than those in the HFD group (Fig. 6f and i, respectively, *P* < 0.05).

### Gyp increased the FXR and SHP mRNA expression

**Fig. 7:**
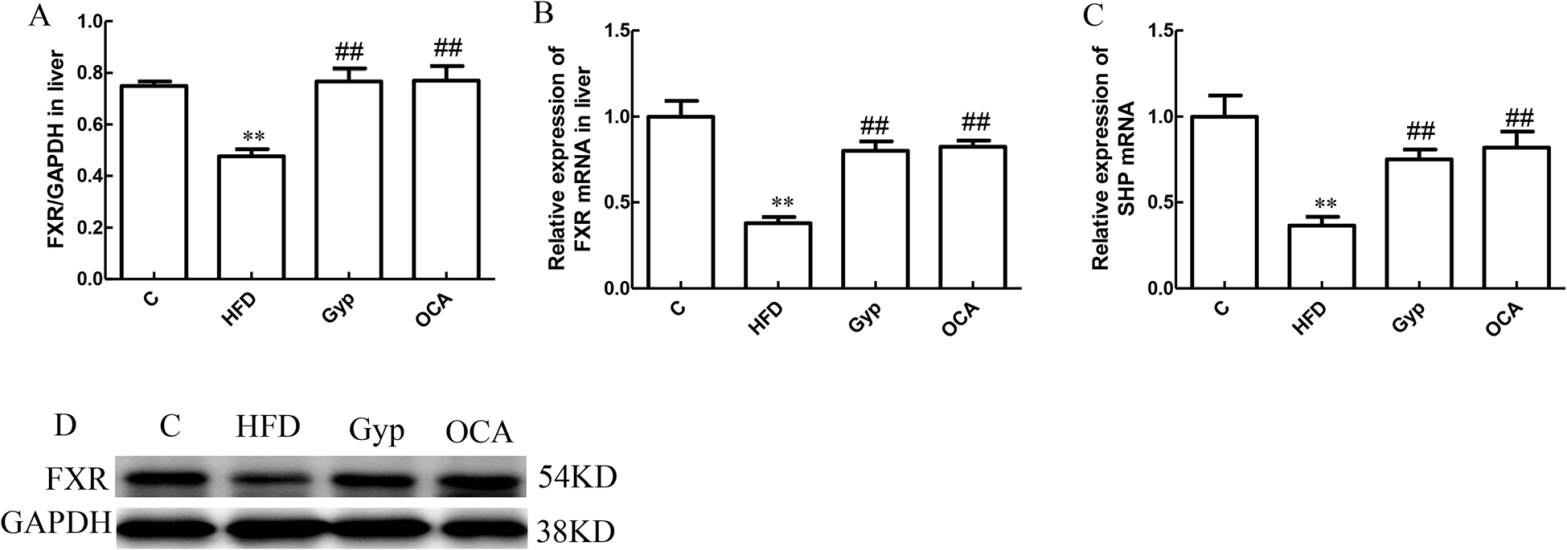
Evaluation of FXR and SHP mRNA expression levels as well as FXR protein levels in liver tissues among the different experimental groups. **a** The relative FXR protein expression level, **b** relative FXR mRNA expression level, and **c** relative SHP mRNA expression level. **d** Autoradiograph image of western blotting with anti-FXR and anti-GAPDH antibodies. **P < 0.01, vs control group; ^##^*P* < 0.01, vs HFD group; ^&^*P* < 0.05, vs OCA group. Values are expressed as mean ± standard deviation, *n* = 3 (protein expression level) or 6 (mRNA expression level). FXR, farnesoid X receptor; SHP, small heterodimer partner. Gyp, gypenosides; OCA, obeticholic acid; C, control; HFD, high-fat diet; Gyp, gypenosides; OCA, obeticholic acid

To gain mechanistic insights into the signaling pathways responsible for the effects of Gyp in NAFLD, we evaluated the mRNA and protein expression of several molecules associated with lipid metabolism in different liver tissues. In the HFD group, the relative mRNA expression of FXR and SHP as well as the FXR protein level were significantly lower than those of the control group (Fig. 7a-d, *P* < 0.01). The relative FXR and SHP mRNA expression levels as well as the FXR protein level were significantly higher in the Gyp-treated and OCA-treated groups (Fig. 7, *P* < 0.01).

### Gyp altered the mRNA expression of SREBP1, SCD1, FASN, PPARα, CPT1, LPL, and MTTP

**Fig. 8:**
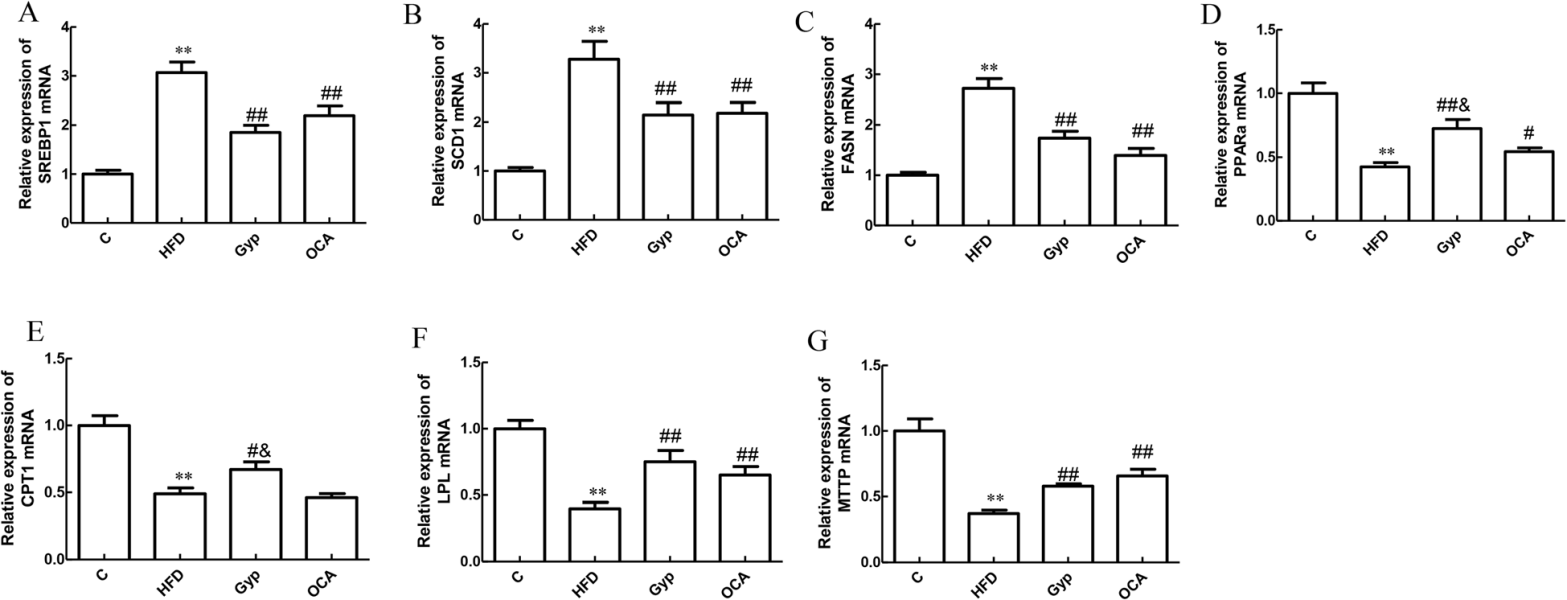
Analysis of the SREBP1, SCD1, FASN, PPARα, CPT1, LPL, and MTTP mRNA levels in liver tissues among the different experimental groups. **a** The relative SREBP1 mRNA expression level, **b** relative SCD1 mRNA expression level, **c** relative FASN mRNA expression level, **d** relative PPARα mRNA expression level, **e** relative CPT1 mRNA expression level, **f** relative LPL mRNA expression level, and **g** relative MTTP mRNA expression level. **P < 0.01, vs control group; ^##^*P* < 0.01, ^#^*P* < 0.05, vs HFD group; ^&^*P* < 0.05, vs OCA group. Data are presented as mean ± standard deviation and are the results of 2 independent experiments, *n* = 6. SREBP1, sterol-regulatory element-binding protein 1; SCD1, stearyl coenzyme A desaturation enzyme 1; FASN, fatty acid synthetase; PPARα, peroxisome proliferator-activated receptor alpha; CPT1, carnitine palmitoyl transferase 1; LPL, lipoprotein lipase; MTTP, microsomal triglyceride transfer protein; C, control; HFD, high fat diet; Gyp, gypenosides; OCA, obeticholic acid

Compared with those in the control group, the SREBP1, SCD1 and FASN mRNA levels were significantly upregulated (Fig. 8a-c, *P* < 0.01) and the PPARα, CPT1, LPL, and MTTP mRNA levels were significantly downregulated in the HFD group (Fig. 8d-g, *P* < 0.01). In the Gyp-treated and OCA-treated groups, the relative mRNA expression levels of SREBP1, SCD1 and FASN were significantly lower than those in the HFD group (Fig. 8a-c*, P* < 0.01). In contrast, the PPARα, CPT1, LPL, and MTTP mRNA levels in the Gyp-treated group (Fig. 8d-g) and the PPARα, LPL and MTTP mRNA levels in the OCA-treated group (Fig. 8d, f and g) were significantly higher than those of the HFD group (*P* < 0.05). Compared to those in the OCA-treated group, the relative mRNA expression levels of PPARα and CPT1 were significantly higher in the Gyp-treated group (Fig. 8d and e, *P* < 0.05).

### Gyp altered the protein levels of SREBP1, SCD1, FASN, PPARα, CPT1, LPL, and MTTP

**Fig. 9:**
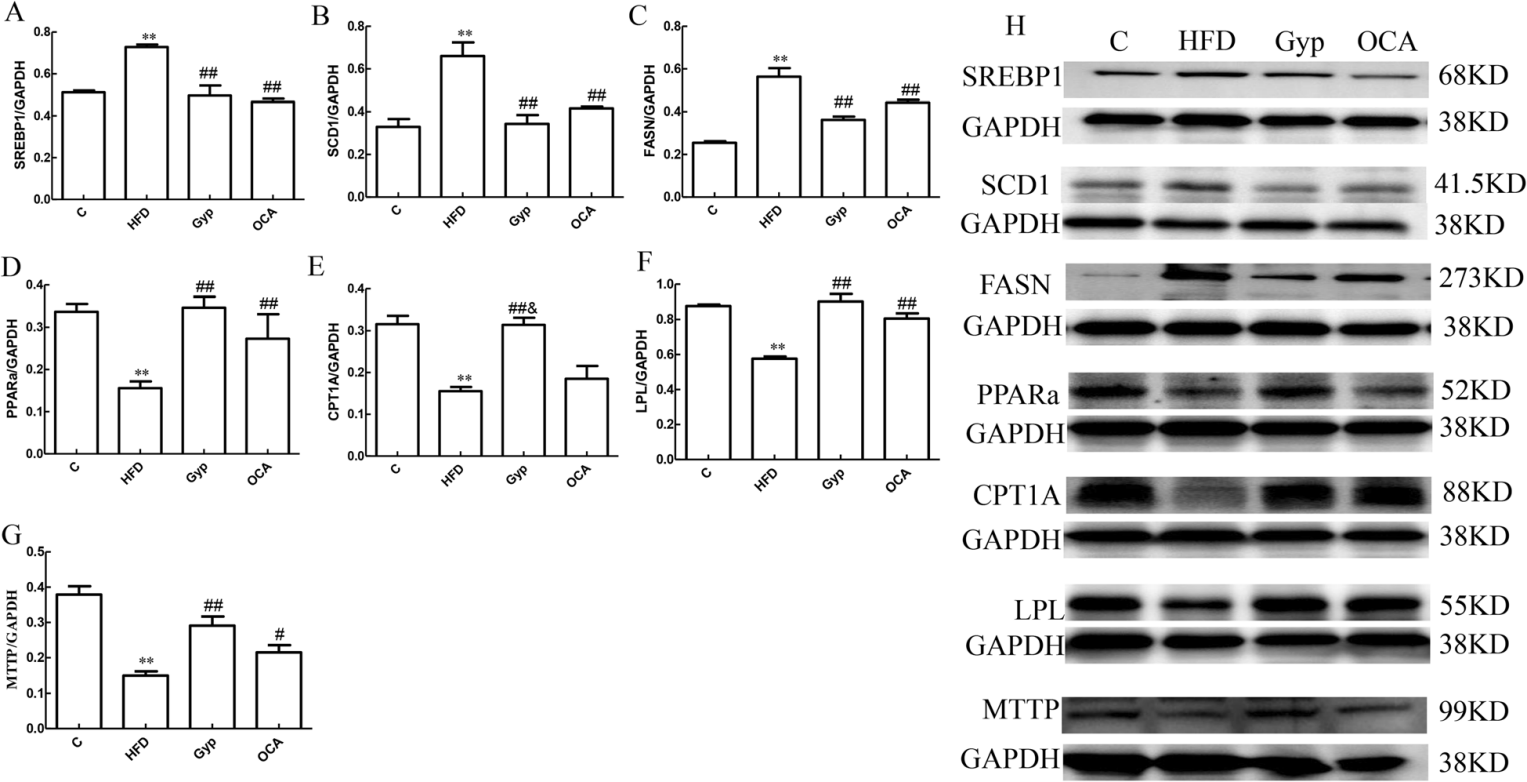
Evaluation of SREBP1, SCD1, FASN, PPARα, CPT1, LPL, and MTTP protein content in liver tissues. **a** Bar chart representing the relative SREBP1 protein expression level, **b** relative SCD1 protein expression, **c** relative FASN protein expression level, **d** relative PPARα protein expression level, **e** relative CPT1A protein expression, **f** relative LPL protein expression level, and **g** relative MTTP protein expression level. **h** Representative autoradiograph image of western blotting with the different antibodies. **P < 0.01, vs control group; ^##^*P* < 0.01, ^#^*P* < 0.05, vs HFD group; ^&^*P* < 0.05, vs OCA group. Data are presented as mean ± standard deviation, *n* = 3. C, control; HFD, high-fat diet; Gyp, gypenosides; OCA, obeticholic acid

The relative expression levels of SREBP1, SCD1 and FASN proteins were significantly higher in the HFD group than the control group (Fig. 9a-c and h, *P* < 0.01). While the relative protein expression levels of PPARα, CPT1, LPL and MTTP were decreased significantly in the HFD group (Fig. 9d-g and h, *P* < 0.01). In the Gyp-treated and OCA-treated groups, the relative expression levels of |SREBP1, SCD1, and FASN proteins were significantly lower than those in the HFD group (Fig. 9a-c and h, *P* < 0.01). The PPARα, CPT1, LPL and MTTP protein levels in the Gyp-treated group and the PPARα, LPL and MTTP protein levels in the OCA-treated group were significantly upregulated compared to the HFD group (Fig. 9, *P* < 0.05).

### Gyp altered FGF15, CYP7A1, and BSEP expression

**Fig. 10:**
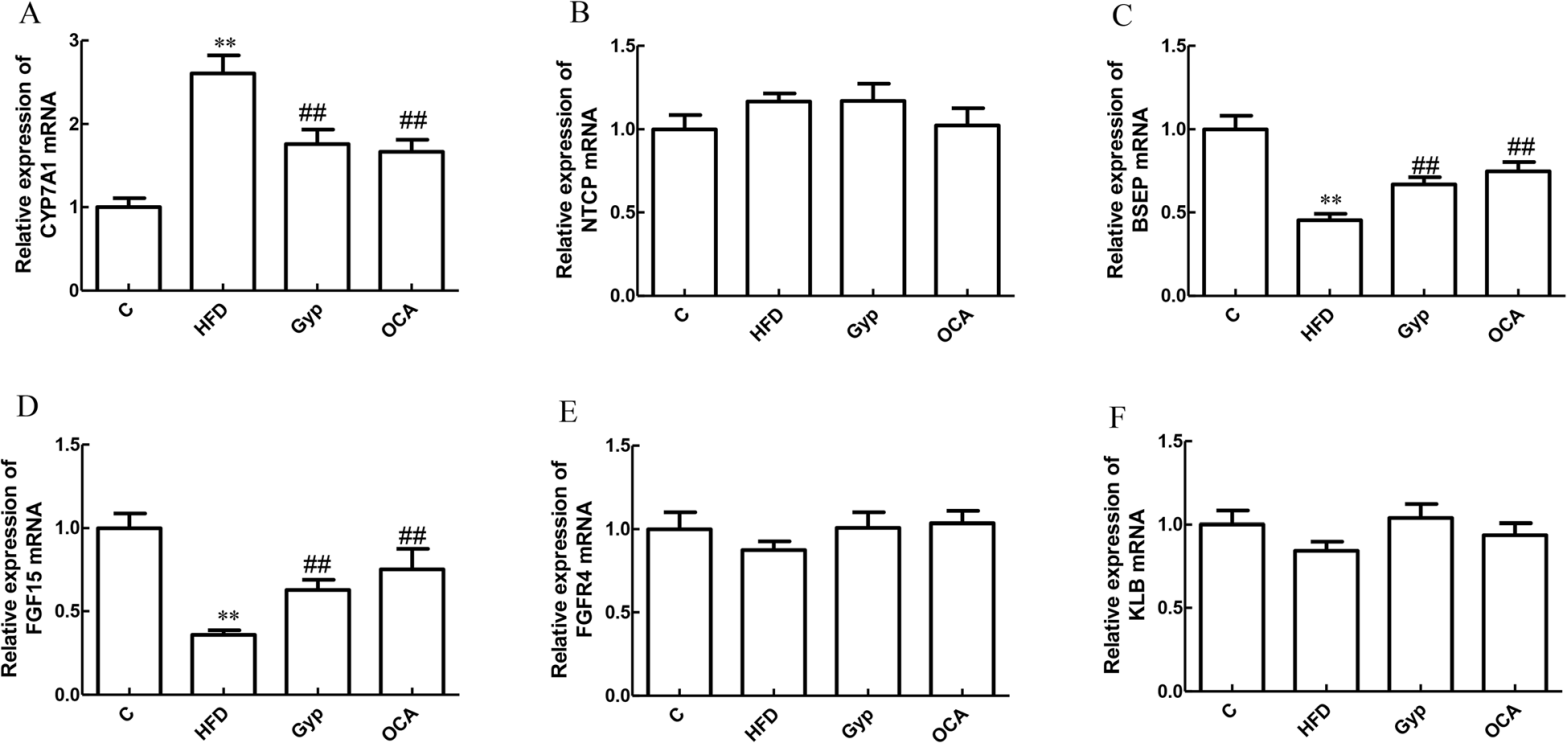
Evaluation of FGF15, FGFR4, KLB, CYP7A1, NTCP, and BSEP mRNA levels in liver tissues among the different experimental groups. **a** Bar chart representing the relative CYP7A1 mRNA expression level, **b** relative NTCP mRNA expression level, **c** relative BSEP mRNA expression level, **d** relative FGF15 mRNA expression level, **e** relative FGF4R expression level, and **f** relative KLB mRNA expression level. **P < 0.01, vs control group; ^##^*P* < 0.01, ^#^*P* < 0.05, vs HFD group. Data are presented as mean ± standard deviation and are the results of 2 independent experiments, *n* = 6. FGF15, fibroblast growth factor 15; FGFR4, fibroblast growth factor receptor 4; KLB, Klotho beta; CYP7A1, cholesterol 7-alpha hydroxy-lase; NTCP, Na + −taurocholate co-transporting polypeptide; BSEP, bile salt export protein; C, control; HFD, high-fat diet; Gyp, gypenosides; OCA, obeticholic acid

Compared to the control group, the relative expression of CYP7A1 mRNA was significantly increased in the HFD group (Fig. 10a, *P* < 0.01). In contrast, the relative mRNA expression of FGF15 and BSEP was significantly lower in the HFD group (Fig. 10c and d, *P* < 0.01). There were no significant differences in the relative FGFR4, KLB, and NTCP mRNA levels between the control group and the HFD group (Fig. 10b, e and f, *P >* 0.05). In the Gyp-treated and OCA-treated groups, the relative FGF15 and BSEP mRNA expression levels were significantly higher than those in the HFD group (Fig. 10c and d, *P* < 0.05), whereas the CYP7A1 mRNA level was significantly lower (Fig. 10a, *P* < 0.01).

### Gyp influenced the protein expression of FGF15, CYP7A1, and BSEP

**Fig. 11:**
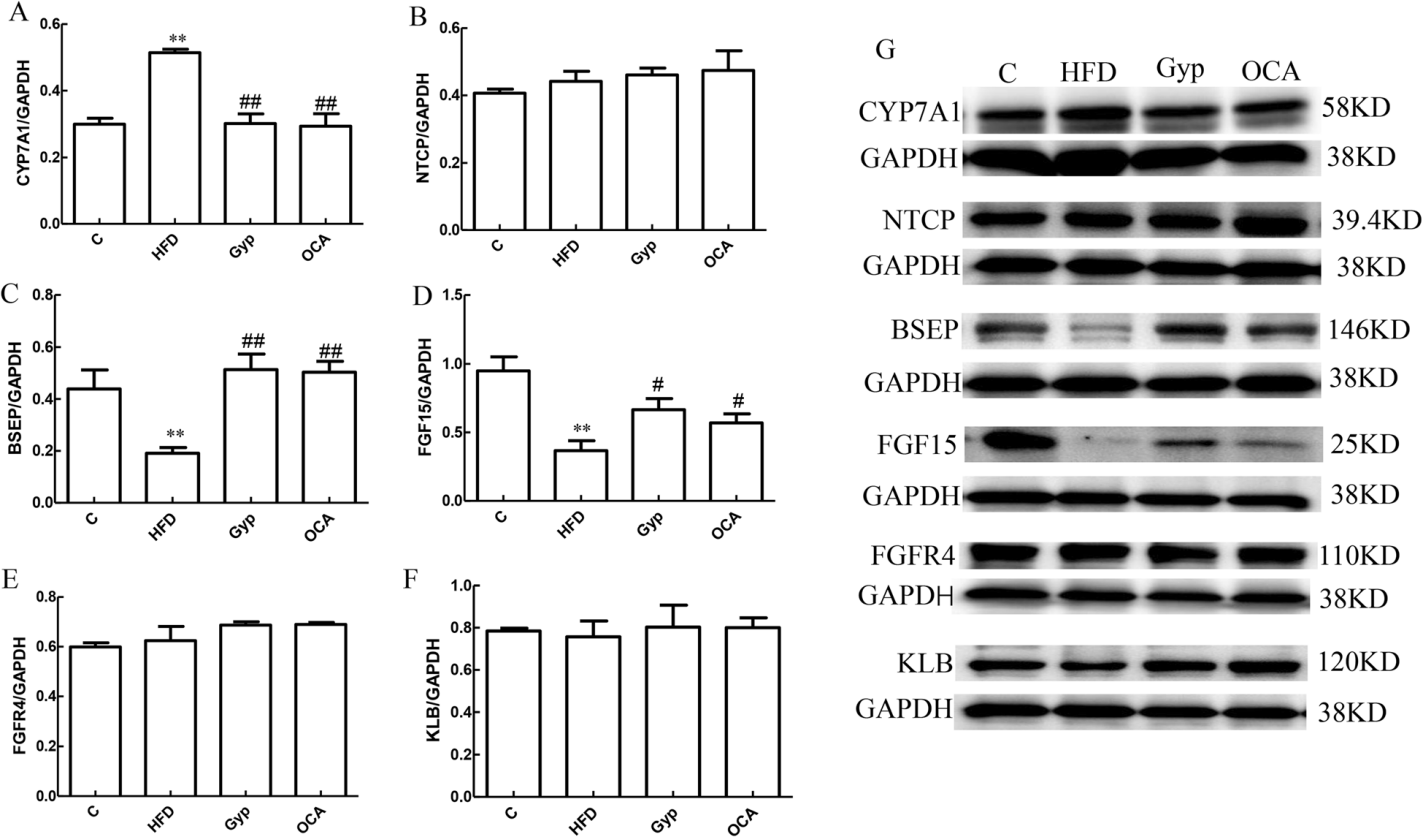
Changes in FGF15, FGFR4, KLB, CYP7A1, NTCP, and BSEP protein content in liver tissues among the different experimental groups. **a** Bar chart representing the relative CYP7A1 protein expression level in liver tissue, **b** relative NTCP protein expression level, **c** relative BSEP protein expression level, **d** relative FGF15 protein expression level, **e** relative FGFR4 protein expression level, and **f** relative KLB protein expression level in liver tissue. **g** Representative autoradiograph image of western blotting with the different antibodies. **P < 0.01, vs control group; ^##^*P* < 0.01, ^#^*P* < 0.05, vs HFD group. Data are presented as mean ± standard deviation, *n* = 3. C, control; HFD, high-fat diet; Gyp, gypenosides; OCA, obeticholic acid

To further investigate the underlying signaling pathway, we analyzed the protein expression levels of FGF15, FGFR4, KLB, CYP7A1, NTCP, and BSEP in liver tissues collected from the different experimental groups (Fig. 11). CYP7A1 protein expression was significantly higher in the HFD group than in the control group (Fig. 11a, *P* < 0.01). Compared with those in the control group, the relative expression levels of FGF15 and BSEP proteins were significantly decreased in the HFD group (Fig. 11c and d, *P* < 0.01). The relative expression levels of CYP7A1 protein were significantly lower in the Gyp-treated and OCA-treated groups than in the HFD group (Fig. 11a, *P* < 0.01). In contrast, the relative expression levels of FGF15 and BSEP proteins were significantly higher in the Gyp-treated and OCA-treated groups (Fig. 11c and d, *P* < 0.05). The FGFR4, KLB, and NTCP protein levels did not differ significantly among the different experimental groups (Fig. 11b, e and f, *P >* 0.05).

## Discussion

NAFLD is a chronic liver disorder that impacts approximately 24% of the adult population worldwide [28]. Steatosis or increased accumulation of intrahepatic TGs is a characterizing feature of NAFLD [29]. Bile acids are crucial for lipid metabolism, and they are exclusively synthesized in hepatocytes [30]. Bile acids are the end products of cholesterol metabolism [31]. Upon entering the intestine, primary bile acids promote lipid digestion and are converted to secondary bile acids by intestinal bacteria. Roughly 95% of intestinal bile acids are absorbed by the intestinal wall and then return to the liver via the portal vein. From the hepatocytes, the absorbed bile acids along with newly formed conjugated bile acids are discharged into the intestinal tract through the bile duct in a process named the enterohepatic circulation of bile acids [32]. Bile acid homeostasis is essential for maintaining normal lipid metabolism [33, 34]. Therefore, accumulating evidence suggests that abnormal bile acid metabolism can lead to the dysregulation of lipid metabolism, which can promote the occurrence and development of NAFLD [35–37]. Moreover, Lu et al. demonstrated that children with NAFLD had altered bile acid profiles and elevated serum TBA levels [38]. In NAFLD, bile acids can improve inflammation and glucolipid metabolism through the regulation of FXR and TGR5 expression and their downstream signaling pathways [35]. Therefore, bile acids and their receptors are crucial for maintaining normal systemic energy metabolism and hepatic lipid metabolism [39].

In this study, we induced NASH in mice by HFD feeding for 14 consecutive weeks. In agreement with previous reports [40], our results demonstrated the successful induction of NASH. We observed histopathological signs of hepatic steatosis along with increased liver TGs and NAS scores in the HFD group. Interestingly, treatment with Gyp or OCA (a FXR agonist) improved the histopathological picture and decreased the liver TG levels as well as the NAS scores. Moreover, we observed that Gyp treatment could significantly reduce the TBA content as well as the aMCA, TaMCA, βMCA, TβMCA TCDCA, CA, GCDCA, GCA and TCA content and significantly increased the alloLCA and βCDCA content in liver tissues from NASH mice, and thus, it promoted hepatic bile acid homeostasis. In agreement, previous research demonstrated that Gyp can alter the liver bile acid homeostasis induced by a HFD [41]. Also, Gyp treatment successfully regulated the adipose thermogenesis and gut microbiota, which were reduced in the process of gaining weight [42]. Moreover, Gyp was shown to treat type 2 diabetes and NAFLD in rats [43]. Altogether, the abovementioned data suggest that Gyp have a beneficial effect on NAFLD. Indeed, our results confirmed the beneficial effect of Gyp on NASH. Notably, the biological impact of Gyp treatment was comparable to that of treatment with the FXR agonist, OCA. Therefore, these results suggest that Gyp and FXR can share a common pathway for the treatment of NASH.

In the normal physiological conditions, FXR requires bile acid activation and mediates negative feedback regulation of bile acid metabolism [44, 45]. FXR is crucial for the regulation of bile acid synthesis, its transportation and maintenance of bile acid homeostasis [46]. Upon activation, FXR can increase the expression of SHP in the liver tissue, thereby inhibiting CYP7A1 expression, regulating NTCP and BSEP expression, and improving bile acid metabolism [47, 48]. Alternatively, FXR can upregulate the expression of FGF15 in the small intestine. The interaction of FGF15 and FGFR4 in the liver, along with KLB can ultimately inhibit the expression of CYP7A1, a key enzyme in bile acid synthesis [49]. Inhibition of the FXR signaling pathway can lead to abnormal bile acid metabolism [50]. In this study, treatment with OCA upregulated the expression of FXR, SHP, FGF15 and BSEP in liver, which was in agreement with previously published results [48]. Furthermore, our results demonstrated that Gyp significantly upregulated the levels of FXR, SHP, FGF15 and BSEP and downregulated the level of CYP7A1 in liver tissues. Therefore, it is plausible to speculate that Gyp can improve bile acid metabolism and maintain bile acid homeostasis by regulating FXR-mediated bile acids metabolic pathways.

FXR plays an important role in the regulation of lipid metabolism [51]. Upon activation, FXR can regulate lipid metabolism and maintain bile acid homeostasis primarily through the following pathways: 1) upregulation of SHP expression and inhibition of sterol-regulatory element-binding protein 1c (SREBP-1c) expression [51, 52]. SREBP-1c is a key regulator of lipogenesis, and its downregulation can significantly decrease the expression of lipogenesis-related genes, like FASN and SCD1, thereby inhibiting lipid deposition [53]; 2) activation of PPARα, upregulation of CPT-1 expression, and promotion of fatty acid oxidation [52]; 3) induction of apolipoprotein CII (ApoCII) expression and inhibition of apolipoprotein CIII (ApoCIII) expression, thereby activating LPL, which promotes TG decomposition [54]; and 4) upregulation of MTTP and very low-density lipoprotein receptor (VLDLR) expression, thereby promoting the transportation and clearance of TG from the liver [55].

The results obtained from this study revealed that Gyp significantly increased the FXR mRNA and protein levels in liver tissues of mice with NASH. Similarly, Gyp treatment increased the SHP level and downregulated the SREBP1 mRNA expression and protein content in liver tissues, thereby decreasing the SCD1 and FASN expression and inhibiting hepatic lipid synthesis. In addition, Gyp treatment upregulated the expression of PPARα and CPT1 in the liver tissues at both the mRNA and protein levels, thereby enhancing fatty acid oxidation. Further, Gyp upregulated LPL and MTTP expression, which led to the promotion of lipid decomposition. Therefore, Gyp are capable of regulating the lipid metabolic pathways by upregulating FXR expression, which can ultimately lead to an improvement in lipid metabolism in NAFLD.

## Conclusions

**Fig. 12:**
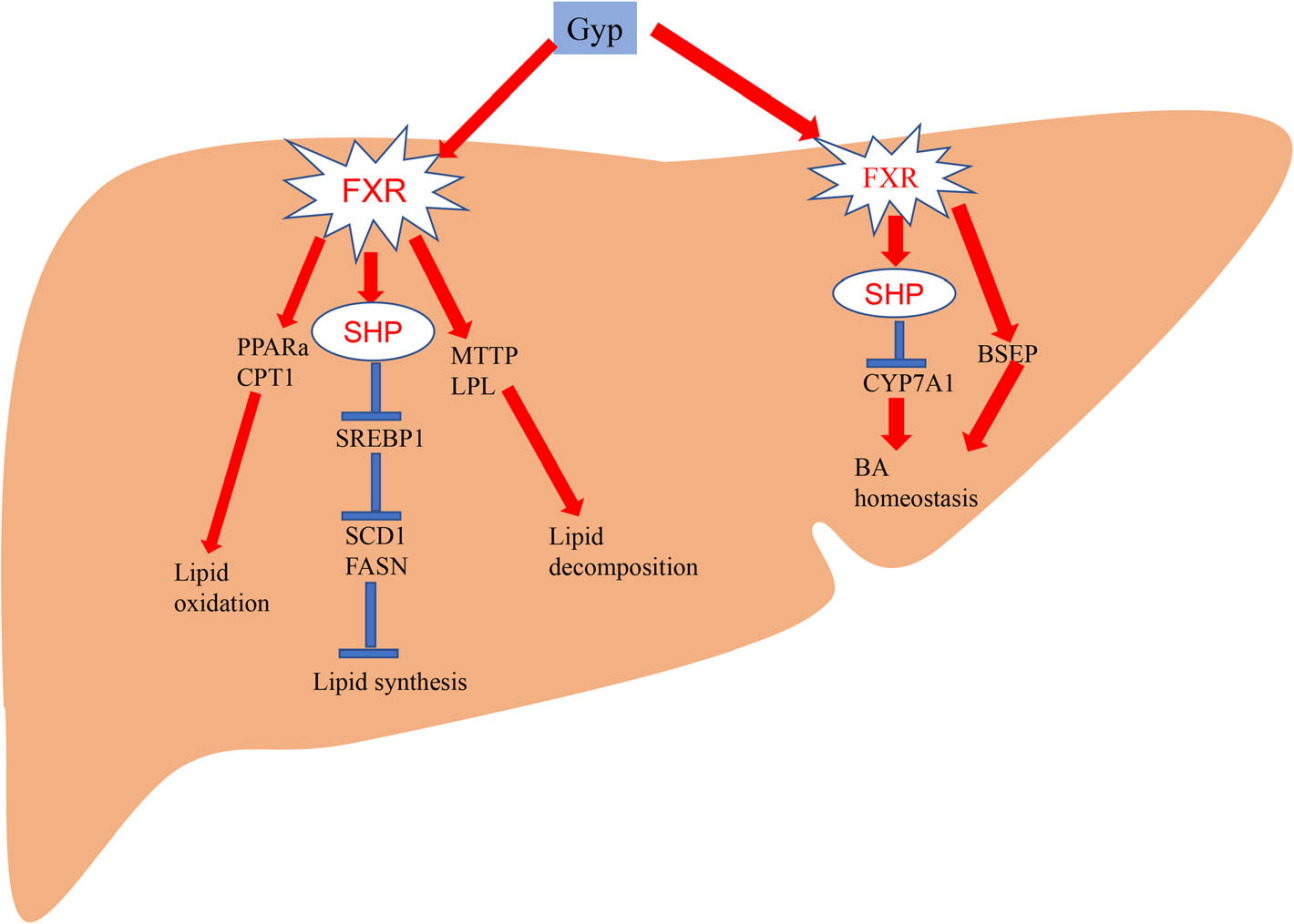
Graphical abstract. Gypenosides treatment upregulates the SHP level and downregulates the SREBP1 level, thereby decreasing SCD1 and FASN expression and inhibiting hepatic lipid synthesis. Meanwhile, gypenosides can upregulate the expression of PPARα and CPT1 and enhance fatty acid oxidation, gypenosides can also upregulate LPL and MTTP expression, which led to the promotion of lipid decomposition. In addition, gypenosides treatment could significantly upregulate FXR-mediated bile acid pathways, thereby maintaining bile acid homeostasis. FXR, farnesoid X receptor; SHP, small heterodimer partner; SREBP1, sterol-regulatory element-binding protein 1; SCD1, stearyl coenzyme A desaturation enzyme 1; FASN, fatty acid synthetase; PPARα, peroxisome proliferator-activated receptor alpha; CPT1, carnitine palmitoyl transferase 1; LPL, lipoprotein lipase; MTTP, microsomal triglyceride transfer protein. CYP7A1, cholesterol 7-alpha hydroxy-lase; BSEP, bile salt export protein

In conclusion, we confirmed that Gyp can significantly reduce the lipid content in the liver and serum, reduce serum ALT and AST activities, improve insulin resistance and ameliorate pathological changes in liver tissues. These results suggest the beneficial impact of Gyp in the treatment of NAFLD. In addition, Gyp treatment could significantly upregulate FXR-mediated bile acid and lipid metabolism pathways, thereby maintaining bile acid and lipid homeostasis. Interestingly, the impact of Gyp on the downstream bile acid and lipid signaling molecules was similar to that of FXR agonist treatment (OCA). Therefore, it is possible to hypothesize that the function of Gyp maybe related to activation of the FXR signaling pathway (Fig. 12).

## Data Availability

The datasets generated and analyzed during the current study are available from the corresponding author on reasonable request.

## Abbreviations

Gyp: Gypenosides
FXR: Farnesoid X receptor
SHP: Small heterodimer partner
SREBP1: Sterol-regulatory element-binding protein 1
SCD1: Stearyl coenzyme A desaturation enzyme 1
FASN: Fatty acid synthetase
PPARα: Peroxisome proliferator-activated receptor alpha
CPT1: Carnitine palmitoyl transferase 1
MTTP: Microsomal triglyceride transfer protein
CYP7A1: Cholesterol 7-alpha hydroxy-lase
FGFR4: Fibroblast growth factor receptor 4
BSEP: Bile salt export protein
NTCP: Na+-taurocholate co-transporting polypeptide
KLB: Klotho beta
FGF15: Fibroblast growth factor 15
LPL: Lipoprotein lipase
HFD: High-fat diet
OCA: Obeticholic acid
ALT: Alanine aminotransferase
AST: Aspartate aminotransferase
FINS: Fasting insulin
FBG: Fasting blood glucose
HOMA-IR: Homeostatic Model Assessment for Insulin Resistance
TG: Triglycerides
TC: Total cholesterol
LDL-C: Low density lipoprotein cholesterol
HDL-C: High density lipoprotein cholesterol
TBA: Total bile acid
αMCA: α-muricholic acid
βMCA: β-muricholic acid
TαMCA: Tauro α-muricholic acid
TβMCA: Tauro β-muricholic acid
alloLCA: allolithocholic acid
TCDCA: Taurochenodeoxycholic acid
CA: Cholic acid
βCDCA: 3β-chenodeoxycholic acid
GCDCA: Glycochenodeoxycholic acid
TCA: Taurocholic acid
GCA: Glycocholic acid
